# Annotations

**Published:** 1910-05-28

**Authors:** 


### Annotations.

### Shakespeare's Medical Information.

One of the interesting features of Shakespeare's plays is the information with regard to medicine in his time which they contain. He evidently knew the names and uses of many drugs, for he mentions aconite, ginger, chamomile, the mints, savory, marjoram, the worts, wormwood, and many others, and it should be remembered that empirical knowledge of drugs and their uses was not monopolised by the medical profession down to the seventeenth century, and even later. Country ladies were expected to keep well-stocked drug closets and to be ready to dispense remedies to their families or dependants. The old village wives gathered herbs and simples and concocted medicaments, and one need only conjecture that Shakespeare as a boy often loitered about while his mother was at her household duties, or sometimes listened to the old women's gossip in or about Stratford, to realise that most of the information in his plays concerning drugs and their uses may easily have been absorbed unconsciously in his early youth. With regard to the descriptions of mental diseases in Shakespeare's plays, the journal of the American Medical Association refers to some documents recently brought to light by Professor Wallace, of the University of Nebraska. These manuscripts show that Shakespeare lived for some years in Muggle Street (now Monkwell Street) in a house opposite to the Barber-Surgeons' Hall, and it is suggested as not being unlikely that he may have come into contact with some of the members of the Guild of Barber-Surgeons, and that some of his medical knowledge may have been obtained from that source.

### Some Recent Fiction.

The current tendency to deal gently with the medical profession in plays and novels is one which has already been remarked in these pages on previous occasions. The same kindness is still to be noted in a recent satire which issues from the pen of Mr. Barry Pain under the title of "The Exiles of Faloo." This novel holds up to ridicule many types of twentieth-century hypocrites, such as the swindling company promoter, the inebriate clergyman, the rascally lawyer, the dissenting newspaper proprietor, and others. Amid this gallery of rogues the doctor stands out conspicuously; for his character is the strongest, his nerve the coolest, of the whole set. Briefly, the scene is laid in a remote Pacific island, used as a refuge by lawbreakers of various descriptions, whither they may retire with their plunder so as to be beyond the reach of justice. In every man's past there has been some fraudulent or criminal act which has made England too hot for him; and in every case but the doctor's this is of some particularly mean or heartless kind. It finally turns out that the latter has left home after serving twelve months' imprisonment for signing an untrue death certificate. Called to attend a man whose wife had suffered the most horrible indignities from him for ten years, the doctor is making a cure when his patient relapses, and dies suddenly with symptoms pointing to arsenical poisoning. Quite certain that the wife has been guilty of administering the fatal dose, he shields her by signing a certificate of death from some natural cause. Three months later the widow in hysterical remorse confesses the truth, and the doctor is wrongly suspected of a guilty intrigue with her and is sentenced to imprisonment. Technically guilty of crime—and the only one of the exiles who has expiated his offence—he is also the only one to whom no serious moral censure attaches at all. It is satisfactory to add that he finally receives his deserts, and that the other exiles also get theirs.

### Administration of the Poison Law.

Except when an irregular sale of poison is brought into public notice by a coroner's inquest, it is a rare occurrence for the police to institute proceedings against a person who sells a poison without fulfilling the legal requirements. The administration of the law relating to the sale of poisons is, in fact, left almost entirely in the hands of the Pharmaceutical Society. Nevertheless, it is open to the police to prosecute anyone who infringes Section xvii. of the Pharmacy Act, 1868; that is to say, sells a poison which is not properly labelled, or fails to make an entry in the poison-book or neglects to observe any of the other legal formalities in selling one of the more potent poisons. On the other hand, if an unqualified person sells a poison (which is an offence under Section xv.) the Pharmaceutical Society is the only body which can recover a penalty. The work entailed in the administration of the penal clauses of the Pharmacy Acts is therefore very considerable, since it is the duty of that body to ensure, as far as possible, that the sale of poisons is conducted in a regular manner, so that the public may be safeguarded. Some particulars of this work are contained in the Society's annual report, which was adopted at the sixty-ninth annual meeting, held on Wednesday of last week. During last year over thirteen thousand cases of alleged infringement were investigated, and proceedings were instituted in 155 cases, in the majority of which the offences consisted in the keeping of open shops for the sale of poisons by unregistered persons or the sale of poisons by such persons. This part of the Society's work cost £835, and in referring to this expenditure the President, in the course of his speech at the annual meeting, said it scarcely seemed fitting that the members of the Society should bear the whole cost of fulfilling this valuable public duty, since the cases taken last year were equivalent to so many arrests of public danger, towards which the general community contributed nothing.

